# Retraction: Knockdown of TUG1 aggravates hypoxia-induced myocardial cell injury *via* regulation of miR-144-3p/Notch1

**DOI:** 10.1039/d1ra90039k

**Published:** 2021-01-26

**Authors:** Laura Fisher

**Affiliations:** Royal Society of Chemistry Thomas Graham House, Science Park, Milton Road Cambridge CB4 0WF UK advances-rsc@rsc.org

## Abstract

Retraction of ‘Knockdown of TUG1 aggravates hypoxia-induced myocardial cell injury *via* regulation of miR-144-3p/Notch1’ by Bo Zhu *et al.*, *RSC Adv.*, 2019, **9**, 22931–22941, DOI: 10.1039/C9RA01311C.

The Royal Society of Chemistry hereby wholly retracts this *RSC Advances* article due to concerns with the reliability of the data. The images in the article were screened by an image integrity expert. All of the western blot bands have very regular, oval shapes that are unlikely to be genuine. Furthermore, the western blots and many other features of the article were found to be unexpectedly similar to western blots and features in a number of other papers with no overlapping authors.

The authors were asked to provide the raw data for this article, but did not respond. Given the significance of the concerns about the validity of the data, and the lack of raw data, the findings presented in this paper are not reliable.

The authors have been informed but have not responded to any correspondence regarding the retraction.

Signed: Laura Fisher, Executive Editor, *RSC Advances*.

Date: 15^th^ January 2021.

## Supplementary Material

